# De Quervain's Disease

**Published:** 2013-07-16

**Authors:** Kushal R. Patel, Kashyap K. Tadisina, Mark H. Gonzalez

**Affiliations:** Department of Orthopedic Surgery, University of Illinois College of Medicine, Chicago

**Keywords:** De Quervain Disease, Stenosing Tenosynovitis, Tendon Entrapment, Tendinopathy

**Figure:**
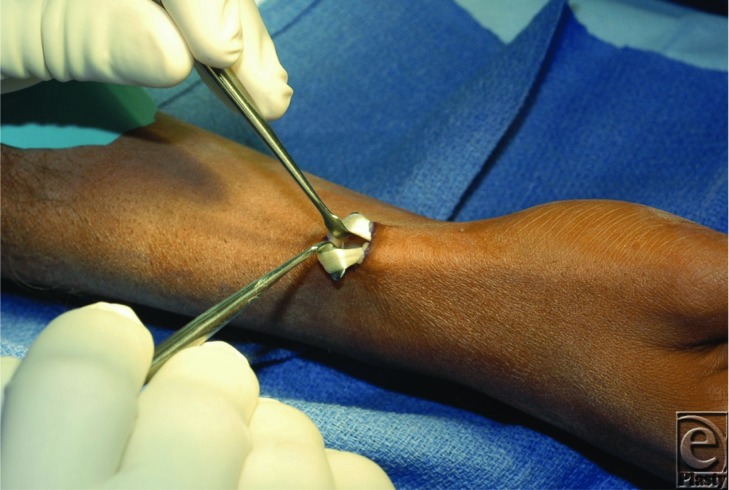


## DESCRIPTION

A 47-year-old woman returns to the clinic with persistent pain, tenderness, and swelling over the thumb side of her wrist despite corticosteroid injections. She is diagnosed with de Quervain's refractory to nonsurgical management and is scheduled for surgical decompression of the first dorsal compartment.

## QUESTIONS

1. **What is the etiology of de Quervain's disease and what should it be differentiated from?**
2. **What are the nonsurgical treatment options and when is surgical decompression indicated for de Quervain's disease?**
3. **What anatomic variations should one be aware of to insure complete decompression?**
4. **What anatomic structure needs to be identified and protected during the surgical procedure?**

## DISCUSSION

De Quervain's disease, also called gamer's thumb or mother's thumb, is a common pathological condition of the wrist. Although the exact mechanism has not been determined, the cause of de Quervain's disease is thought to be due to thickening of the synovial sheath containing the extensor pollicis brevis (EPB) and abductor pollicis longus (APL) tendons, which leads to irritation of the muscles, causing pain and swelling over the radial side of the wrist in patients along with an increased difficulty in gripping objects.^1^ Studies have shown de Quervain's tendon sheaths to be thickened and fibrosed with nodularities, but no inflammatory changes being present. More commonly found in perimenopausal and pregnant women, de Quervain's disease has been linked to overuse, although no clear evidence has supported this notion.^2^^,^^3^ This presentation is not unique to de Quervain's disease, as pain and discomfort over the radial part of the distal wrist can also be a sign of intersection syndrome, osteoarthritis of the thumb carpometacarpal joint, or Wartenberg's syndrome. A thorough history and a series of physical examination maneuvers, including the Finkelstein test, can help differentiate between these causes.

A number of nonsurgical treatments are available to treat de Quervain's tenosynovitis with main options including corticosteroids, nonsteroidal anti-inflammatory drugs, and splinting. There is limited evidence showing any benefits to nonsteroidal anti-inflammatory drugs or splinting except in those patients with minimal symptoms.^4^ Clinically, corticosteroids are most commonly used to treat de Quervain's disease. Studies report success rates of 62% to 93% with various corticosteroid formulations.^4^ Correct technique involves infiltration of corticosteroid into the tendon sheath and into EPB subsheath if present. A second corticosteroid injection may be performed at 4 to 6 weeks after the first; however, subsequent repeat injections are not recommended.^5^ The risks associated with corticosteroid injection include thinning of skin due to fat necrosis of subcutaneous tissue, depigmentation of skin in darker skinned individuals around the injection site, and tendon rupture with repeated injections. Surgical decompression is reserved for patients who have failed conservative treatment.

It is important to understand the anatomical variations found within the compartment as failure to do so can result in inadequate surgical decompression and persistent pain. Anatomical studies have described the variations seen in tendon structure and organization of EPB and APL as well as the tendon sheath. Extensor pollicis brevis has been found to be absent in 2% to 7% of the population and is found to be generally thinner than APL.^5^^,^^6^ In one study of 66 cadaver limbs, the APL was found to have single tendinous slip in 9, 2 slips in 46, 3 slips in 9, and 4 slips in 2.^6^ The first dorsal compartment is found to be subdivided, partially or completely, by a septum forming 2 distinct tunnels in 20% to 60% of specimens studied.^6^ The EPB is found in the more ulnarly located tunnel created by the septation and the APL with its varying number of slips in the more radially located tunnel.^5^ In such a scenario, either tunnel or both are capable of becoming stenotic and producing symptoms, thus it is imperative to identify and excise the subsheath at time of surgery.

Grossly, the radial styloid, Lister's tubercle, and the scaphoid tubercle have been found to be reliable landmarks for the location of the first dorsal compartment. Decompression is accomplished via a transverse incision that is made over the area of the first dorsal compartment. Sharp dissection through the subcutaneous tissue is generally avoided to prevent injury to the superficial branches of the radial nerve. The sheath is incised on the dorsoulnar aspect making sure to excise any subsheath present for complete decompression. An ulnar incision of sheath provides an adequate retaining wall to prevent volar subluxation, whereas a radial incision or complete excision of the tendon sheath is avoided because it can result in painful volar subluxation.^5^ If 3 clearly separate tendon slips are identified (1 EPB, 2 APL), the expected pattern in 70% of cases, there is a low likelihood that another tendon slip is being overlooked because of the presence of a subsheath.^6^ Complications associated with de Quervain's release include injury to the superficial branches of the radial nerve with resulting formation of a painful neuroma and superficial radial nerve entrapment.^5^ Thus, meticulous dissection and gentle retraction of the nerve is necessary to avoid stretching or cutting the nerve. If surgery fails to provide pain relief, consideration should be given to an unreleased subsheath or other conditions such as carpometacarpal joint, interphalangeal joint arthritis, or intersection syndrome.

## Figures and Tables

**Figure 1:**
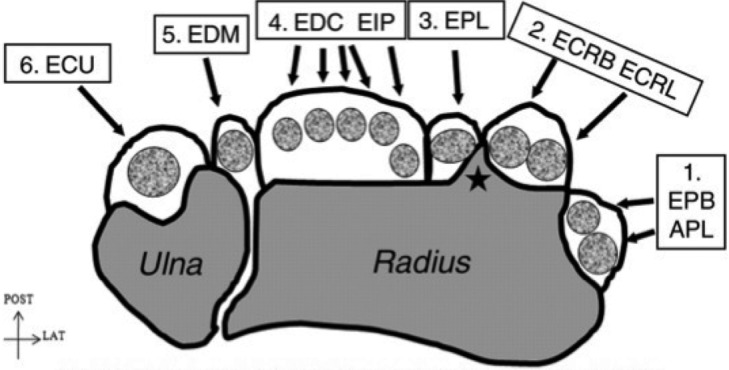
Dorsal extensor tendons of the wrist in their six (1 -6) separate compartments. APL indicates abductor pollicis longus; EPB, extensor pollicis brevis; ECRB, extensor carpi radialis brevis; ECRL, extensor carpi radialis longus; ECU, extensor carpi ulnaris; EDC, extensor digitorum communis; EDM, extensor digiti minimi; EIP, extensor indicis proprius. Rousset P, Vuillemin-Bodaghi V, Laredo J-D, Parlier-Cuau C. Anatomic variations in the first extensor compartment of the wrist: accuracy of US. *Radiology*. 2010;257:427-33. Copyright 2010 RSNA.
